# Effect of Malaria and *Schistosoma mansoni* Coinfection on Selected Biochemical Profiles among Patients Attending Selected Health Institutions at Dembiya, Northwest Ethiopia

**DOI:** 10.1155/2024/9992233

**Published:** 2024-03-21

**Authors:** Wagaw Abebe, Wossenseged Lemma, Yalewayker Tegegne, Amare Mekuanint, Abebe Yenesew, Adane Derso

**Affiliations:** ^1^Department of Medical Laboratory Sciences, College of Health Sciences, Woldia University, Woldia, Ethiopia; ^2^Department of Medical Parasitology, School of Biomedical and Laboratory Sciences, College of Medicine and Health Sciences, University of Gondar, Gondar, Ethiopia; ^3^Department of Clinical Chemistry, School of Biomedical and Laboratory Sciences, College of Medicine and Health Sciences, University of Gondar, Gondar, Ethiopia; ^4^Department of Medical Laboratory Sciences, College of Medicine and Health Sciences, Debre Markos University, Debre Markos, Ethiopia

## Abstract

**Background:**

Malaria and schistosomiasis are infectious diseases that cause biochemical abnormalities. Malaria and *Schistosoma mansoni* coinfection causes exacerbations of health consequences and comorbidities. The study area is found in Ethiopia, where coinfection of malaria and *S. mansoni* is common. However, there is limited data on the biochemical profiles of patients coinfected with malaria and *S. mansoni* schistosomiasis in the study area. Hence, this study aimed to assess the effect of malaria and *S. mansoni* schistosomiasis coinfection on selected biochemical profiles.

**Methods:**

An institutional-based comparative cross-sectional study was conducted from March 30 to August 10, 2022. Using a convenient sampling technique, 70 participants (35 cases and 35 controls) were enrolled in the study. *Schistosoma mansoni* was detected in stool samples using the wet mount and the Kato Katz method. To detect *Plasmodium*, both thick and thin blood films were prepared and stained with Giemsa. The blood sample was processed for the analysis of biochemical profiles. All data were analyzed using SPSS version 25. A *p* value of less than 0.05 was considered statistically significant.

**Results:**

The mean values of alanine aminotransferase and aspartate aminotransferase (37.1 U/L and 41.9 U/L, respectively) in coinfected participants were significantly higher than in the healthy control participants (17.4 U/L and 22.0 U/L, respectively) (*p* < 0.05). Also, the median values of creatinine, total bilirubin, and direct bilirubin (1.51 mg/dL, 2.35 mg/dL, and 0.91 mg/dL, respectively) in coinfected participants were significantly higher than in the healthy control participants (0.85 mg/dL, 0.42 mg/dL, and 0.12 mg/dL, respectively) (*p* < 0.05). However, median values of total protein (4.82 g/dL) and mean values of glucose (66.6 mg/dL) in coinfected participants were significantly lower than in the healthy control participants (total protein (7.64 g/dL) and glucose (91.9 mg/dL)) (*p* < 0.05). The results of biochemical profiles in healthy participants were significantly different from those with light, moderate, and heavy *S. mansoni* infection intensity in malaria and *S. mansoni* coinfection (*p* < 0.05). *Schistosoma mansoni* infection intensity had a positive correlation with biochemical profiles except for total protein and glucose, which correlated negatively in coinfected participants (*p* > 0.05).

**Conclusion:**

Biochemical profiles in coinfection were significantly changed as compared to healthy individuals. As a result, biochemical profile tests should be utilized to monitor and manage coinfection-related problems, as well as to reduce coinfection-related morbidity and death.

## 1. Background

Malaria is an infectious disease caused by protozoan parasites of the genus *Plasmodium* and transmitted by female *Anopheles* mosquitoes [1]*. Plasmodium falciparum* and *P. vivax* are the main cause of malaria infection worldwide [2]. According to the World Health Organization (WHO, 2023), malaria caused 249 million cases in 85 malaria endemic countries in 2022 and an increase of 5 million cases compared with 2021 in the world [3].

Malaria parasites go through a hepatocyte developmental stage. Sporozoites produced from the salivary gland of a mosquito must effectively target and penetrate hepatocytes [4]. These parasites replicate in the red blood cells of their human host following an initial replication phase in the liver. These parasite erythrocytic replication cycles result in the typical disease symptoms, including fever, anemia, and eventually lead to organ failure and patient death [5]. Sequestration of erythrocytes with mature forms of the parasite in the deep vascular beds of vital organs is the major pathologic hallmark of severe malaria. *Plasmodium falciparum* malaria frequently causes life-threatening complications such as cerebral malaria, renal failure, hepatic dysfunction, jaundice, abnormal bleeding, and severe anemia [6].

Furthermore, malaria induces biochemical changes within the host [7]. Sporozoites invade hepatocytes in the liver stage, causing organ congestion, sinusoidal blockage, and cellular inflammation. Hepatocyte changes can result in the leakage of parenchyma and membrane enzymes into the general circulation [8]. Due to this, malaria causes biochemical abnormalities such as high bilirubin, elevated aspartate aminotransferase (AST), elevated alanine aminotransferase (ALT), and high creatinine, which increase the risk of disease complications [9]. Malaria also affects almost all organ systems, but the most dangerous complication of severe malaria is acute kidney disease [10]. Acute renal failure is characterized by pathological diseases in the kidneys that cause anemia, jaundice, hypoglycemia, acidosis, and coma [11]. The existence of hemoglobinopathies, nutritional status, demographic factors, and the degree of malaria immunity can all affect the physicochemical properties of red blood cells that have been infected with malaria parasites [12, 13].

Schistosomiasis is an infection caused by a digenetic intravascular parasite that inhabits the venous portal mesenteric system in humans [14]. *Schistosoma mansoni*, *S. haematobium,* and *S. japonicum* are the species that infect human beings among the *Schistosoma* species [15]. Schistosomiasis, the second-most common tropical disease in the sub-Saharan region, continues to cause significant morbidity and mortality in developing countries [16]. Based on World Health Organization (WHO, 2020), it caused 24 000 deaths and 2.5 million disability adjusted life years [17].

The type of *Schistosoma* species and the severity of infection both influence the clinical presentation and pathology of schistosomiasis [18]. The mesenteric plexus is a habitat for *S. mansoni* and leads to intestinal or hepatosplenic schistosomiasis which affects the intestine, liver, and spleen [16]. *Schistosoma mansoni* infection's pathophysiological effects are mostly related to the development of granulomas brought on by the host cell-mediated immune response to soluble antigens released by parasite eggs trapped in the hepatic and intestinal vessels of infected hosts [14]. These egg-induced granulomas cause liver failure, which leads to protein synthesis impairment and increment in ALT and AST levels [19].

Furthermore, schistosomiasis causes stunted growth, cognitive impairment, anemia, impaired aerobic capacity, and death [16]. The level and duration of exposure, the intensity of the infection, concurrent infections, nutritional status, parasite strain, and genetic predisposition are some of the factors that can influence the clinical symptoms and severity of schistosomiasis disease [20].

Malaria and *S. mansoni* infections cause public health and socioeconomic development challenges [21]. And also, morbidity and mortality related to malaria and *S. mansoni* infection remain a major concern in the world. Coinfection of these parasites is frequent in sub-Saharan Africa (SSA), where over 90% of these diseases occur due to a large geographic overlap [22–24]. Both diseases have been associated with poverty, and factors that contribute to their spread include a low socioeconomic status, inadequate sanitation, limited access to potable water, a lack of education, and a lack of awareness [25, 26].

In coinfection of malaria and *S. mansoni,* alters the balance between T helper cell 1 (Th1) and Th2 immune responses and lowering malaria immunological control. Schistosome eggs have immunomodulatory potential by inducing the alternative activation of macrophages and regulatory T-cell expansion. This may reduce the Th1 response, but it may increase Th2 immune responses, increasing the risk of early clinical malaria. And also, acute infection with schistosomes increases the levels of Th1 cytokines, which increases the severity of malaria disease [27–31]. In addition, this immunologic response alteration may result in a reduction of the effectiveness of malaria treatment in malaria and *S. mansoni* coinfection [32]. However, research on the effect of coinfection with malaria and *S. mansoni* on biochemical profile is still limited. Studying malaria and *S. mansoni* coinfection's effect on biochemical profile is important to reducing different problems like impaired protein synthesis and liver fibrosis which are related to those coinfections. Also, well-informed alterations in biochemical profile in malaria and *S. mansoni* coinfection enable the clinician to establish reliable diagnosis and therapeutic interventions. Therefore, the current study assessed the effects of malaria and *S. mansoni* coinfection on biochemical profile.

## 2. Methods and Materials

### 2.1. Study Design, Area, Period, and Population

An institutional-based comparative cross-sectional study was conducted from March 30 to August 10, 2022, at Dembiya Primary Hospital, Chuahit Health Center, and Abrija Health Center, which are located within the Central Gondar Administrative Zone, Amhara Regional State. Dembiya Primary Hospital, Chuahit Health Center, and Abrija Health Center are found in Dembiya district. The southern part of the district is bordered by Lake Tana. Rivers within this district include Angereb and Derma. These rivers serve as sources of water for bathing, washing clothes, and other domestic and recreational purposes. They may contain the major sources of malaria and *S. mansoni* infections. As reported by the District Health Bureau, malaria and *S. mansoni* are endemic in the study area.

The study populations were malaria and *S. mansoni* coinfected and healthy control participants. Women who were pregnant, people who had multiple intestinal parasite infections, people who were receiving antiretroviral therapy, people who had a history of chronic diseases like hypertension, cardiac disease, diabetes mellitus, and chronic renal disease, people who were positive for hepatitis B and hepatitis C viruses, smokers, and people who used alcohol excessively were excluded from the study.

### 2.2. Sample Size Determination and Sampling Technique

The sample size was determined based on rules of thumb that have been recommended by van Voorhis and Morgan: 30 study subjects per group are required to detect real differences, which leads to about 80% power [33]. Thus, 70 study participants (35 infected by both malaria and *S. mansoni* and 35 healthy participants; sex and age match control) were enrolled in the study. A convenient sampling technique was used to select study participants. All clinically suspected individuals for malaria and/or *S. mansoni* infection and those who fulfilled the inclusion criteria and presented themselves to Dembiya selected health institutions outpatient department (OPD) were enrolled in the case group. Also, participants who were microscopically positive for both malaria and *S. mansoni* and gave blood and stool samples and whose age is five years and above were enrolled in the study as study participants. All health-seeking individuals attending the voluntary council and testing (VCT) clinic of Dembiya selected health institutions and who fulfilled the inclusion criteria were enrolled in the control group. In addition, participants who were microscopically negative for both malaria and *S. mansoni* and gave blood and stool samples, and whose age is five years and above were enrolled in the study as healthy control participants.

### 2.3. Data Collection Procedures

#### 2.3.1. Questionnaire Survey

Sociodemographic characteristics of study participants were collected using a semistructured questionnaire prepared in Amharic language. The questionnaire was initially written in English language and translated into Amharic language. Sociodemographic data was collected by trained medical laboratory personnel and nurses. Trained clinicians who work at Dembiya selected health institutions OPD and VCT clinics assessed the clinical information and patient history. After identifying individuals who were eligible for the study, the volunteer study participants were linked to medical laboratory personnel for blood and stool sample collection.

### 2.4. Sample Collection and Laboratory Examination

#### 2.4.1. Microscopic Detection of Plasmodium

Six microliters and 2 *μ*l of capillary blood were collected and placed separately on a tiny glass slide for preparing thick and thin blood films, respectively, by experienced laboratory technicians. Both thick and thin, were prepared and air dried. Absolute methanol was used to fix thin blood films, and both films were stained for 10 minutes with a 10% Giemsa working solution. An experienced malaria microscopist read both thin and thick blood films with a 100x objective lens and examined 100 microscopic fields to rule out the presence or absence of malaria parasites. A trained malaria microscopist confirmed the disparity findings [34].

#### 2.4.2. Microscopic Detection of Schistosomes

Each study participant provided a single stool specimen measuring approximately 1 g. The sample was collected in a clean, dry, and leak-proof container labeled with a unique identification number. Each stool specimen was analyzed using the direct wet mount technique, followed by Kato-Katz slides prepared on a template containing 41.7 mg. Eggs counted for *S. mansoni* were recorded and afterward changed into eggs per Gram (EPG) of stool by multiplying by a factor of 24 [35]. Finally, infection intensity was classified as light (1–99 EPG), moderate (100–399 EPG), and heavy (400 and above EPG) using WHO standards [36].

#### 2.4.3. Blood Sample Collection and Examination

Three milliliters (3 ml) of venous blood were collected by experienced blood collectors using a sterile disposable plastic syringe after cleaning the venous puncture site with 70% alcohol. The collected blood sample was transferred into test tubes. The collected venous blood was transferred into a nonanticoagulated tube and allowed to clot on the bench top. Blood was centrifuged at 2,500 revolutions per minute for four minutes, and serum was separated and stored in an Eppendorf tube at −20°C until processed. Then, serum was analyzed using Fully Auto Chemistry Analyzer (COBAS C 311) for serum levels of ALT, AST, creatinine, glucose, total bilirubin, direct bilirubin, and total protein [37].

### 2.5. Data Quality Control

Data collectors took appropriate training to maintain data quality. Quality control was performed by re-reading all slides by an expert laboratory technologist to ensure the accuracy of *Plasmodium* and *Schistosoma* detection. Standard operating procedures and manufacturer instructions were strictly followed throughout the procedures, and all reagents were stored and prepared according to the manufacturer's instructions.

### 2.6. Data Management and Analysis

Data were coded and entered into the EpiData (v3.1) statistical software, and exported to Statistical Package for the Social Sciences (SPSS) version 25 for analysis. The homogeneity of variance was checked using Levene's statistics. The skewness, kurtosis, and Shapiro–Wilk normality tests were used for checking the distribution of continuous variables, and it revealed that ALT, AST, and glucose were normally distributed and total protein, creatinine, total bilirubin, and direct bilirubin were not normally distributed for each group. One-way analysis of variance (ANOVA), independent *t*-test, and post hoc Tukey Honest Significant Difference (HSD) tests were used for the comparison of normally distributed biochemical profiles between groups. Biochemical profile values of ALT, AST, and glucose were normally distributed. Nonparametric tests, Kruskal–Wallis H, and Mann–Whitney *U* tests were used for comparison of abnormally distributed biochemical profiles between groups. Biochemical profile values like creatinine, total bilirubin, direct bilirubin, and total protein were abnormally distributed. The correlation of variables was assessed using Pearson and Spearman's rank correlation analysis techniques. For each group, the result was provided as the mean and standard deviation (SD) for normally distributed data. Similarly, the result was provided as the median and interquartile range **(**IQR) for abnormally distributed data for each group. A *p* value of less than 0.05 was considered statistically significant in all statistical analyses.

## 3. Results

### 3.1. Sociodemographic Characteristics of Study Participants

A total of 70 study participants were recruited from Dembiya Primary Hospital 36 (51.4%), Chuahit Health Center 18 (25.7%), and Abrija Health Center 16 (22.9%). Participants from rural and urban areas were 36 (51.4%) and 34 (48.6%), respectively. Among 70 study participants, 35 were malaria and *S. mansoni* coinfected, and 35 were healthy control participants. The prevalence of malaria and *S. mansoni* coinfection was higher in males (18, 51.4%) than females (17, 48.6%), and higher in the age group of 5–14 years (11, 31.4%) than those other age groups (15–24 years, 8 (22.9%), 25–34 years, 8 (22.9%), 35–44 years, 5 (14.3%), and >44 years, 3 (8.6%)) (Table 1).

### 3.2. Intensity of *Schistosoma mansoni* Infection

**Figure 1:**
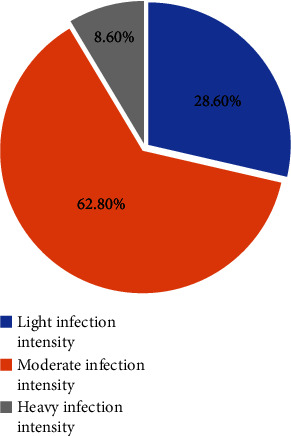
Intensity of *S. mansoni* infection in malaria and *S. mansoni* coinfected participants at Dembiya selected health institutions, 2022.

The mean of the EPGs of stool from malaria and *S. mansoni*-coinfected participants was 224.9. The means of the EPG in males and females were 246.3 and 184.0 in malaria and *S. mansoni*-coinfected participants, respectively. From a total of 35 malaria and *S. mansoni-*coinfected participants 10 (28.5%), 22 (62.9%), and 3 (8.6%) had light, moderate, and heavy *S. mansoni* infection intensities, respectively (Figure 1).

### 3.3. Biochemical Profiles among Study Participants

**Figure 2:**
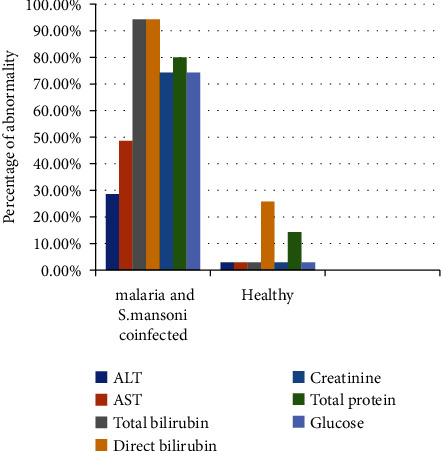
Prevalence of abnormal biochemical profiles of study participants at Dembiya selected health institutions, 2022.

The percentages of elevated ALT, AST, total bilirubin, direct bilirubin, and creatinine were higher in malaria and *S. mansoni*-coinfected participants than in healthy control participants. However, the percentages of lowered total protein and glucose were higher in malaria and *S. mansoni-*coinfected participants than in healthy control participants. Among malaria and *S. mansoni*-coinfected participants, 10 (28.6%), 17 (48.6%), 33 (94.3%), 33 (94.3%), and 26 (74.3%) had elevated ALT, AST, total bilirubin, direct bilirubin, and creatinine, respectively. Similarly, 25 (71.4%), 17 (51.4%), 2 (5.7%), 2 (5.7%), 7 (20.0%), 7 (20%), and 8 (22.9%) of the malaria and *S. mansoni*-coinfected participants had normal ALT, AST, total bilirubin, direct bilirubin, creatinine, total protein, and glucose, respectively. On the other hand, 28 (80%) and 26 (74.3%) of the malaria and *S. mansoni*-coinfected participants had decreased total protein and glucose, respectively (Figure 2).

### 3.4. Comparison of Biochemical Profiles among Study Participants

In malaria and *S. mansoni*-coinfected participants, 37.1 (7.17) IU/L, 41.9 (8.83) IU/L, and 66.6 (14.0) mg/dL were the mean (SD) values of ALT, AST, and glucose, respectively (Table 2). In addition, 1.51 (0.56) mg/dL, 2.35 (1.07) mg/dL, 0.91 (0.96) mg/dL, and 4.82 (2.32) g/dL were the median (IQR) values of creatinine, total bilirubin, direct bilirubin, and total protein, respectively, in the malaria and *S. mansoni*-coinfected participants (Table 3).

The Mann–Whitney *U* test showed significantly higher median values for creatinine, total bilirubin, and direct bilirubin in malaria and *S. mansoni*-coinfected participants compared to healthy control participants (*p* < 0.05). However, the total protein median value was significantly lower in malaria and *S. mansoni*-coinfected participants compared to healthy control participants (*p* < 0.05). An independent *t*-test revealed significantly higher mean values for ALT and AST but a lower mean value of glucose in malaria and *S. mansoni*-coinfected participants compared to healthy control participants (*p* < 0.05).

### 3.5. Biochemical Profiles across Different Level of *Schistosoma mansoni* Intensity Infection among Study Participants

The mean (SD) values of ALT, AST, and glucose were 17.4 (8.6) IU/L, 22.0 (0.93) IU/L, and 91.9 (12.8) mg/dL, respectively, in healthy control participants. The mean values of ALT, AST, and glucose were 35.7 (6.26) IU/L, 40.2 (7.27) IU/L, and 68.2 (14.8) mg/dL in moderate infected participants and 43.8 (4.81) IU/L, 50.6 (0.86) IU/L, and 60.9 (6.18) mg/dL in heavy *S. mansoni*-infected participants, respectively (Table 4). Similarly, the median (IQR) values of creatinine, total bilirubin, direct bilirubin, and total protein in healthy control participants were 0.85 (0.17) mg/dL, 0.42 (0.36) mg/dL, 0.12 (0.13) mg/dL, and 7.64 (1.61) g/dL, respectively. In addition, 1.43 (0.70) mg/dL, 2.48 (1.12) mg/dL, 0.89 (1.15) mg/dL, and 5.14 (3.3) g/dL were the median values of creatinine, total bilirubin, direct bilirubin, and total protein, respectively, in light *S. mansoni*-infected participants (Table 5).

One-way ANOVA revealed that the mean (SD) values of ALT, AST, and glucose showed a significant difference among healthy controls, light, moderate, and heavy *S. mansoni*-infected participants (*p* < 0.05 in each). Likewise, the Kruskal–Wallis H-test also revealed that the median (IQR) of creatinine, total bilirubin, direct bilirubin, and total protein showed significant differences among healthy controls, light, moderate, and heavy *S. mansoni*-infected participants (*p* < 0.05 in each). The mean values of ALT and AST were significantly lowered, but glucose was significantly higher in healthy control participants compared to those with light, moderate, and heavy *S. mansoni* infection intensity (*p* < 0.05). The median values of creatinine, total bilirubin, and direct bilirubin were significantly lowered, but total protein was significantly higher in healthy participants compared to those with light, moderate, and heavy *S. mansoni* infection intensity in malaria and *S. mansoni* coinfection (*p* < 0.05). Total bilirubin was also significantly higher in patients with heavy *S. mansoni* infection compared to those with moderate *S. mansoni* infection (*p* < 0.05). However, the mean values of ALT, AST, and glucose were not significantly different between light and moderate, light and heavy, and moderate and heavy *S. mansoni* infections. Also, creatinine, direct bilirubin, and total protein median values did not differ significantly between light and moderate, light and heavy, and moderate and heavy *S. mansoni* infection (*p* > 0.05) (Table 6).

### 3.6. Correlation of Intensity of *Schistosoma mansoni* Infection with Biochemical Profiles

In malaria and *S. mansoni-*coinfected participants, Pearson correlation analysis showed that the number of *S. mansoni* EPG of stool had been nonsignificantly and positively correlated with biochemical profiles (ALT and AST) (Pearson correlation coefficients *r* = 0.115 and 0.088, respectively; *p* > 0.05). However, glucose was nonsignificant, and negatively correlated with the number of *S. mansoni* EPG of stool in malaria and *S. mansoni* coinfected participants (Pearson correlation coefficient *r* = −0.068; *p* > 0.05). Similarly, Spearman's rank order correlation analysis showed that the number of *S. mansoni* EPG of stool had been nonsignificantly and positively correlated with biochemical profiles (creatinine, total bilirubin, and direct bilirubin) (Spearman's rho correlation coefficient *r* = 0.136, 0.65, and 0.055, respectively; *p* > 0.05) in malaria and *S. mansoni*-coinfected participants. But the total protein of malaria and *S. mansoni*-coinfected participants was nonsignificantly and negatively correlated with the number of *S. mansoni* EPG of stool (Spearman's rho correlation coefficient *r* = −0.096; *p* > 0.05) (Table 7).

## 4. Discussion

Malaria and *S. mansoni* infections cause public health and socioeconomic development challenges [21]. The present study aimed to investigate the effects of malaria and *S. mansoni* coinfection on the biochemical profiles at Dembiya Selected Health Institutions, Northwest Ethiopia.

The study found that participants with both malaria and *S. mansoni* coinfection had significantly higher mean values of ALT and AST, as well as median values of creatinine, total bilirubin, and direct bilirubin compared to the healthy control group. Conversely, participants who were coinfected with *S. mansoni* and malaria had mean glucose levels and median total protein levels that were considerably lower than those of healthy control participants. This finding was similar to studies conducted in Ethiopia that found significantly higher values of ALT, AST, direct bilirubin, and lower values of glucose and total protein in the *S. mansoni* monoinfected participants as compared to healthy participants [39, 40]. In addition, this finding was similar with a study conducted in Western Kenya that found significantly higher median value of ALT in the malaria and *S. mansoni*-coinfected participants as compared to healthy participants [23]. This finding was also consistent with a study conducted in Yemen, which found significantly higher levels of AST, ALT, total bilirubin, and direct bilirubin in malaria patients compared to healthy individuals [9]. A study carried out in India provided additional evidence similar to this finding [41]. This could be due to the coinfection of malaria and *S. mansoni*, which can cause more severe and chronically debilitating morbidity than a single parasite infection. This may cause a high degree of intravascular hemolysis of parasitized red blood cells, which leads to a high level of bilirubin in malaria and *S. mansoni*-coinfected participants [42]. Also, this elevation of ALT, AST, total bilirubin, and direct bilirubin might be due to dysfunction of the liver in malaria and *S. mansoni* coinfection. Malaria and *S. mansoni* infection may cause jaundice and hepatomegaly, which lead to an elevation of liver enzymes and bilirubin [43–46]. Low levels of glucose and total protein might be due to glucose utilization and impaired protein synthesis in malaria and *S. mansoni* coinfection [47]. However, this finding was in disagreement with studies conducted in Nigeria [48] and India [49], which found significantly higher values of ALT, AST, creatinine, total bilirubin, direct bilirubin, and lower levels of total protein in the healthy control group as compared to the malaria mono-infected group. This variation might be due to the difference in study participants, the intensity of infection, level and duration of exposure, concurrent infections, nutritional status, parasite strain, genetic predisposition, or the combined malaria and *S. mansoni* infection effect.

Additionally, in this study, the mean values of ALT and AST and the median values of creatinine, total bilirubin, and direct bilirubin were significantly lower in healthy control participants compared to those with light, moderate, and heavy *S. mansoni* infection intensity in malaria and *S. mansoni* coinfection. However, median values of total protein and mean values of glucose were significantly higher in healthy control participants compared to those with light, moderate, and heavy *S. mansoni* infection intensity in malaria and *S. mansoni* coinfection (*p* < 0.05). Correspondingly, the median value of total bilirubin was significantly elevated in heavy *S. mansoni* infection intensity compared with those with moderate *S. mansoni* infection intensity in malaria and *S. mansoni* coinfection. This finding was comparable with a study conducted in Ethiopia [39]. This imbalance in biochemical profiles might be due to the impairment of organs like the liver and kidney as a result of the progression and increment of *S. mansoni* intensity. Since organ-specific morbidity is brought on by the accumulation of *S. mansoni* eggs. The severity of hepatomegaly usually correlates with the intensity of infection and can be exacerbated by chronic exposure to *Plasmodium* species [50–52].

Furthermore, in this study, findings were confirmed by Pearson correlation analysis, where the number of *S. mansoni* EPG of stool had a positive correlation with biochemical profiles (ALT and AST) and a negative correlation with glucose of malaria and *S. mansoni*-coinfected participants. In addition, these findings were confirmed by Spearman's rank order correlation analysis, where the number of *S. mansoni* EPG in stool had a positive correlation with biochemical profiles (creatinine, total bilirubin, and direct bilirubin) and a negative correlation with the total protein in malaria and *S. mansoni*-coinfected participants. The possible scientific reason for this correlation was due to schistosomiasis pathology, which often positively correlates with the intensity of the infection, as indicated by excreted egg counts. This might be due to the major causes of schistosomiasis complications caused by eggs, which may lodged within the host organs such as the liver, spleen, and kidneys, they leading to abnormal biochemical profiles [53]. This study had limitations. In this study, participants who were five years of age and older were recruited, excluding children who were under five years of age and pregnant women. Also, we did not enumerate parasite infections by molecular techniques such as polymerase chain reaction; we only determined parasite density by microscopy technique.

## 5. Conclusions and Recommendations

In the current study, biochemical profiles in malaria and *S. mansoni*-coinfected participants were significantly altered compared to healthy control participants. Since mean values of ALT and AST were higher, but glucose was lower in malaria and *S. mansoni*-coinfected participants compared to healthy participants. In addition, the median values of total bilirubin, direct bilirubin, and creatinine were higher, but the total protein was lower in malaria and *S. mansoni*-coinfected participants compared to healthy participants.

The mean values of ALT, AST, glucose, and the median values of creatinine, total bilirubin, direct bilirubin, and total protein in healthy participants were significantly different from those with light, moderate, and heavy *S. mansoni* infection intensity among malaria and *S. mansoni*-coinfected participants. *Schistosoma mansoni* infection intensity had a positive correlation with biochemical profiles except for total protein and glucose, which correlated negatively in coinfected participants.

Therefore, screening patients for biochemical profile changes has the greatest role in treating malaria and *S. mansoni*-coinfected patients. Also, assessing biochemical profile changes in patients with malaria and *S. mansoni* coinfection is an important step toward reducing malaria and *S. mansoni* coinfection associated morbidity and mortality. Similarly, assessing biochemical profiles is important for the diagnosis of infections and to monitor the progression of the disease. In general, further studies need to be conducted to elucidate the possible alteration of biochemical profiles as a consequence of malaria and *S. mansoni* coinfection in different epidemiological settings, which include children under the age of 5 years and pregnant women. Finally, we would like to recommend that patients be screened and treated for malaria and *S. mansoni* coinfection-associated biochemical profile abnormalities to prevent biochemical disorders.

## Figures and Tables

**Table 1:** Sociodemographic characteristics of the study participants at Dembiya selected health institutions, 2022.

Sociodemographic characteristics	*S. mansoni* and malaria coinfection		Healthy	
	(*n* = 35)		(*n* = 35)	
	Frequency	%	Frequency	%
Sex				
Male	18	51.4	16	45.7
Female	17	48.6	19	54.3
Age				
5–14	11	31.4	7	20
15–24	8	22.9	11	31.4
25–34	8	22.9	10	28.6
35–44	5	14.3	4	11.4
>44	3	8.6	3	8.6
Residence				
Urban	16	45.7	18	51.4
Rural	19	54.3	17	48.6
Occupation				
Government employee	5	14.3	8	22.9
Nongovernment employee	2	5.7	4	11.4
House wife	3	8.6	4	11.4
Student	13	37.1	11	31.2
Daily laborer	3	8.6	2	5.7
Farmer	7	20	4	11.4
Merchant	2	5.7	2	5.7
Educational status				
Illiterate	7	20	4	11.4
Can read and write	4	11.4	6	17.1
Primary school	14	40	4	11.4
Secondary school	3	8.6	2	5.7
College/university	1	2.9	13	37.1
Diploma and above	6	17.1	6	17.1
Family size				
1–3	12	34.3	19	54.3
4–6	7	20	9	25.7
7–9	11	31.4	4	11.4
>9	5	14.3	3	8.6

**Table 2:** Independent *t* test for ALT, AST, and glucose among study participants at Dembiya selected health institutions, 2022.

Profiles	*S. mansoni* and malaria coinfected participants	Healthy control participants	*p* value
	Mean (SD)	Mean (SD)	
ALT (IU/L)	37.0 (7.2)	17.4 (8.6)	<0.001
AST (IU/L)	41.9 (8.8)	22.0 (7.9)	<0.001
Glucose (mg/dL)	67.6 (16.1)	89.1 (25.2)	<0.001

**Table 3:** Comparison of biochemical profiles among study participants at Dembiya selected health institutions, 2022.

Profiles	*S. mansoni* and malaria coinfected participant	Healthy control participants	*p* value
	Median (IQR)	Median (IQR)	
Creatinine (mg/dL)	1.51 (0.6)	0.85 (0.2)	<0.001
Total bilirubin (mg/dL)	2.35 (1.1)	0.42 (0.4)	<0.001
Direct bilirubin (mg/dL)	0.91 (1.0)	0.12 (0.1)	<0.001
Total protein (g/dL)	4.74 (1.6)	7.51 (1.2)	<0.001

**Table 4:** Comparison of ALT, AST, and glucose among study participants at Dembiya selected health institutions, 2022.

Profiles	Healthy	Light	Moderate	Heavy	*p* value
	Mean (SD)	Mean (SD)	Mean (SD)	Mean (SD)	
ALT (IU/L)	17.4 (8.6)	38.0 (8.8)	35.7 (6.26)	43.8 (4.81)	<0.001
AST (IU/L)	22.0 (0.93)	43.0 (11.71)	40.2 (7.27)	50.6 (0.86)	<0.001
Glucose (mg/dL)	91.9 (12.8)	64.7 (14.1)	68.2 (14.8)	60.9 (6.18)	<0.001

**Table 5:** Biochemical profiles at different level of *Schistosoma mansoni* infection intensity among study participants at Dembiya selected health institutions, 2022.

Profiles	Healthy	Light	Moderate	Heavy	*p* value
	Median (IQR)	Median (IQR)	Median (IQR)	Median (IQR)	
Creatinine (mg/dL)	0.85 (0.17)	1.43 (0.70)	1.48 (0.72)	1.71 (0.97)	<0.001
Total bilirubin (mg/dL)	0.42 (0.36)	2.48 (1.12)	2.23 (0.84)	2.79 (1.14)	<0.001
Direct bilirubin (mg/dL)	0.12 (0.13)	0.89 (1.15)	0.91 (0.91)	1.02 (0.95)	<0.001
Total protein (g/dL)	7.64 (1.61)	5.14 (3.3)	4.87 (2.27)	3.86 (1.02)	<0.001

**Table 6:** Multiple pairwise comparisons of biochemical profiles among study participants at Dembiya selected health institutions, 2022.

Profiles	^A^vs^B^	^A^vs^C^	^A^vs^D^	^B^vs^C^	^B^vs^D^	^C^vs^D^
ALT	<0.001^*∗*^	<0.001^*∗*^	<0.001^*∗*^	0.874	0.674	0.345
AST	<0.001^*∗*^	<0.001^*∗*^	<0.001^*∗*^	0.804	0.505	0.179
Creatinine	<0.001^*∗*^	<0.001^*∗*^	0.004^*∗*^	0.951	0.176	0.277
Total bilirubin	<0.001^*∗*^	<0.001^*∗*^	0.004^*∗*^	0.113	0.499	0.037^*∗*^
Direct bilirubin	<0.001^*∗*^	<0.001^*∗*^	0.005^*∗*^	0.393	0.735	0.452
Total protein	<0.001^*∗*^	<0.001^*∗*^	0.005^*∗*^	0.807	0.612	0.452
Glucose	<0.001^*∗*^	<0.001^*∗*^	0.002^*∗*^	0.901	0.974	0.815

*Note*. ^A^Healthy control; ^B^light intensity of infection; ^C^moderate intensity of infection; ^D^heavy intensity of infection. ^*∗*^Significant at *p* < 0.05.

**Table 7:** Correlation of *S. mansoni* infection intensity with biochemical profiles among malaria and *S. mansoni* coinfected participants at Dembiya selected health institutions, 2022.

Profiles	Pearson correlation coefficient	Spearman's rho correlation coefficient	*p* value
ALT	0.115		>0.05
AST	0.088		>0.05
Glucose	−0.068		>0.05
Creatinine		0.136	>0.05
Total bilirubin		0.065	>0.05
Direct bilirubin		0.055	>0.05
Total protein		−0.096	>0.05

## Data Availability

The data used to support the findings of this study are available from the corresponding author upon reasonable request.

## Abbreviations

ALT: Alanine aminotransferase
ANOVA: Analysis of variance
AST: Aspartate aminotransferase
EPG: Eggs per gram
G: Gram
IQR: Interquartile range
ML: Milliliters
SD: Standard deviation
Th1: T helper cell 1
VCT: Voluntary council and testing
WHO: World Health Organization.

## Additional Points

*Definitions*. Healthy individuals were microscopically negative for both malaria and *S. mansoni*. Also, selected biochemical profiles were ALT, AST, creatinine, glucose, total bilirubin, direct bilirubin, and total protein. The normal value of ALT, AST, total bilirubin, direct bilirubin, creatinine, total protein, and glucose were 0–41 U/L, 0–40 U/L, 0–1.2 mg/dl, 0–0.2 mg/dl, 0.7–1.2 mg/dl, 3.5–5.2 g/dl, and 74–109 mg/dl, respectively [54].
